# Comprehensive health status and health-related quality of life of children at diagnosis of high-risk neuroblastoma: a multicentric pilot study

**DOI:** 10.1016/j.htct.2024.05.014

**Published:** 2024-09-07

**Authors:** Karina Viani, William Furlong, Vicente Odone Filho, Mariana dos Santos Murra, Juliana Moura Nabarrete, Elena Ladas, Ronald Duncan Barr

**Affiliations:** a Instituto de Tratamento do Câncer Infantil (ITACI), Hospital das Clínicas, Faculdade de Medicina, Universidade de São Paulo. São Paulo, Brazil; b Centre for Health Economics and Policy Analysis, McMaster University, Hamilton, Canada; c Hospital Israelita Albert Einstein, São Paulo, Brazil; d Hospital de Amor Infantojuvenil de Barretos, Hospital de Amor. Barretos, Brazil; e Department of Pediatrics, Division of Hematology/Oncology/Stem Cell Transplantation, Columbia University Irving Medical Center, New York, NY, USA; f McMaster Children's Hospital, Hamilton, Canada

**Keywords:** Quality of life, Neuroblastoma, Pediatrics

## Abstract

**Background:**

Neuroblastomas account for 8–10 % of all cancer diagnoses among children. Most patients present with advanced, high-risk disease and 90 % are less than five years old. The burden of morbidity and mortality is high and is quantifiable by measures of health-related quality of life (HRQL). Measuring quality of life in under five-year-old children is a particular challenge that has been met with the development of the Health Utilities Pre-School (HuPS) instrument. Quality of life studies in children with cancer are scarce in low- and middle-income countries and are usually conducted at a single center, thus limiting any conclusions drawn. This pilot study aimed to assess the health-related quality of life of children at the time of diagnosis of high-risk neuroblastomas.

**Method:**

This prospective cross-sectional multicentric study assessed the quality of life of children with high-risk neuroblastoma. The Health Utilities Pre-School instrument was applied to under five-year-olds, and the related Health Utilities Index Mark 3 instrument to over five-year olds.

**Main Results:**

Eleven patients participated in this study. There was a high burden of morbidity at diagnosis, often equating to severe disability, indicative of states of health with scores worse than being dead in two under five-year-old children.

**Conclusion:**

The results of the current study will help to set research priorities for subsequent investigations and provide a basis to improve supportive care for children with high-risk neuroblastoma.

## Introduction

In Brazil, cancer represents the leading cause of death by disease of children and adolescents aged from 1 to 19 years old.^1^ Neuroblastomas account for 8–10 % of all cancer diagnoses among children and are the commonest form of cancer within the first year of life.^2^ The overall incidence is 1 per 7000 live births, yielding about 500 new cases of neuroblastoma diagnosed annually in Brazil.^3^ Unfortunately, most patients present with advanced, high-risk disease^4^ and 90 % are younger than five years old. The burden of morbidity and mortality is high and is quantifiable by measures of health-related quality of life (HRQL).

HRQL has been defined as “the value assigned to duration of life as modified by the impairments, functional states, perceptions, and social opportunities that are influenced by disease, treatment or policy.”^5^ Measures of HRQL may be specific, e.g. pain in arthritis, or generic. The latter has the advantage of broad applicability in clinical and general populations, affording the opportunity for comparisons. This group includes preference-based measures which use comprehensive multi-attribute health status classification systems and related preference functions to calculate utility scores for single attributes (domains or dimensions) and a multi-attribute utility score for overall HRQL. This strategy allows an integration of morbidity and mortality in utility scores, contributing to the estimation of quality-adjusted life years (QALYs) and cost-utility analyses, a special form of cost-effectiveness analysis used in economic evaluations.^6^ The Health Utilities Index (HUI) is a generic, multi-attribute, preference-based system employed to describe health status and HRQL; it is able to capture both attribute-specific and overall HRQL.^7^ In Brazil, it has been used in a sample of long-term cancer survivors in childhood,^8^ after translation and cultural adaptation,^9^ and among a sample of teenagers undergoing cancer treatment.^10^ There are few reports of HRQL in children with neuroblastomas although HUI has been used in a national cross-sectional study in Canada of survivors of advanced neuroblastoma who had undergone autologous hematopoietic stem cell transplantation.^11^

Measuring HRQL in under five-year-old children, the most important developmental phase in the human lifespan,^12^ is a particular challenge which has been met by the development of the Health Utilities Pre-School (HuPS) tool,^13^ an instrument that is based on the HUI3 system.^14^ These systems provide individual health state vectors, each of which consists of one level for each attribute. The HuPS classification system defines 27 unique comprehensive health states (6 vision, 5 hearing, 4 speech, 4 ambulation, 4 emotion, and 4 pain and discomfort levels of morbidity). HuPS and HUI3 scores of HRQL are commensurate and continuous, permitting aggregation of these measurements for the assessment of subjects <5 and ≥5 years of age. Validated disability categories of these HRQL scores have been reported.^15^ Inter-rater reliability (parent versus clinician) with HuPS is excellent; the intra-class correlation is 0.79.^13^ Recently, a translated and culturally adapted version of HuPS was published in Brazilian Portuguese.^16^ This has allowed the current study in pre-school aged children at the time of diagnosis of high-risk neuroblastomas as a component of a larger study of children with this disease conducted together with members of the International Initiative for Pediatrics and Nutrition (IIPAN) which is headquartered at Columbia University Irving Medical Center in New York.

Studies of HRQL in children with cancer are scarce in low- and middle-income countries (LMICs) and most were conducted in a single center, thereby limiting their conclusions.^17^ This represents a significant gap in the literature and creates obstacles in determining the optimal standards of care for this patient population. An alternative approach is to measure disability-adjusted life years (DALYs) which are based on disease^18^ rather than on the patient, as with HRQL. More than 80 % of children with cancer studied in respect to DALYs are from LMICs.^19^

Prior to clinical studies on therapeutic interventions, it is important to better comprehend the effect of high-risk neuroblastoma on quality of life to heighten awareness of disease-related morbidity and its treatment and, thus, optimize supportive care. Therefore, this pilot study aimed to assess the HRQL of children at the time of the diagnosis of high-risk neuroblastoma.

## Material and methods

### Study design

This is a prospective cross-sectional multicentric study exploring HRQL in children at the time of diagnosis of high-risk neuroblastoma. Patients were recruited from three pediatric oncology centers in São Paulo State: Instituto de Tratamento do Câncer Infantil (ITACI) and Hospital Israelita Albert Einstein (HIAE) in the city of São Paulo, and Hospital de Amor (HA) in Barretos.

The inclusion criterion was:

- Diagnosis of high-risk neuroblastoma according to the International Neuroblastoma Staging System (INSS).^4^

The exclusion criteria were:

- patients who had previously received anti-cancer treatment;
- under two-year-old children;
- the inability to assess the patient within 48 hours of the beginning of treatment.

### Subjects

Study subject (patient) ‘groups’ are defined by the HRQL instrument and age since these two factors are fully confounded.

### Procedures

This study was approved by the ethics committees of the three institutions. Upon identification of an eligible participant, the participant/family was approached about the participant's enrollment in the study. If the eligible participant/family agreed, and consent and assent (the latter for child ≥7 years of age) were obtained, the forms were appropriately signed as the study procedures were explained to the patient/family.

Assessment of HRQL occurred within 48 h of starting treatment.

### Demographic and clinical data abstraction

Demographic variables were collected from medical records, including the date of birth, sex, city/state/country of residence, the date of diagnosis and race.

### Measures

Questionnaires referring to the patient's previous week were handed to the patient's parent. HuPS was used for under five-year-old children,^16^ and HUI3 for over 5-year-olds.^9^ The disability categories for HuPS and HUI3 reflect scores of HRQL.

### Data management and analysis

All data collection was entered into REDCap, which is a secure database accessible from any location. Attributes and HRQL scoring scales are common across HuPS and HUI3. The eight shared attributes are vision, hearing, speech, ambulation, dexterity, emotion, cognition and pain and discomfort. Attribute level 1 (i.e., no morbidity) is common across HuPS and HUI3. Primary analyses are combined across both <5 and ≥5 groups (*n* = 11). Secondary detailed analyses of attribute levels other than level 1 are of the HuPS group, not of ‘All’ group, because most attribute levels are not common across HuPS and HUI3 systems.

Health state vectors are composites of one level for each attribute. Overall HRQL utility scores range from −0.28 to 1.00 in HuPS and −0.36 to 1.00 in HUI3 with 1.00 equivalent to perfect health, 0.00 equivalent to being dead and negative scores equivalent to states of health worse than being dead.^13^

Statistical analyses are used to describe the distribution of measurements: frequencies (e.g., percent of sample) for ordinal-scale variables (e.g., levels of attributes) and summary statistics for interval-scale variables (e.g., mean of HRQL scores).

### Results

Eleven patients participated in the study, 8 (72.7 %) <5 years of age and 3 (27.3 %) ≥5 years of age. Table 1 summarizes the demographic characteristics of the sample.

**Table 1:** Summary of demographic characteristics.

**Variable**	**<5 years old**	**≥5 years old**	**All patients**
Sex n (%)			
Male	4 (50.0)	2 (66.7)	6 (54.5)
Female	4 (50.0)	1 (33.3)	5 (45.5)
Age (years)			
mean	2.7	6.4	3.7
median	2.7	6.4	3.2
(range)	(1.5–4.1)	(5.1–7.7)	(1.5–7.7)
Race n (%)			
White	5 (62.5)	1 (33.3)	6 (54.5)
Mixed	3 (37.5)	2 (66.7)	5 (45.5)
Treatment Center n (%)			
ITACI	3 (37.5)	3 (100.0)	6 (54.5)
HIAE	1 (12.5)	0 (0.0)	1 (9.1)
HA	4 (50.0)	0 (0.0)	4 (36.4)

ITACI: Instituto de Tratamento do Câncer Infantil; HIAE: Hospital Israelita Albert Einstein; HA: Hospital de Amor (HA).

### Primary analyses

One subject had morbidities in two attributes and the other 10 children (>90 %) had morbidities in three or more attributes (Table 2). The mean overall HRQL utility score for both groups of subjects separately was indicative of severe disability with considerable variation in the younger age group in which the score for two subjects was indicative of moderate disability; two subjects had negative scores indicative of health states worse than being dead (Table 3).

**Table 2:** Number of attributes with morbidity (attribute levels > 1), by subject group.

**Number of attributes with levels >1**	**Study group - n (%)**		
	**<5 years old**	**≥5 years old**	**All patients**
0	0 (0)	0 (0)	0 (0)
1	0 (0)	0 (0)	0 (0)
2	1 (12.5)	0 (0)	1 (9.1)
3	3 (37.5)	2 (66.7)	5 (45.5)
4	2 (25)	1 (33.3)	3 (27.3)
5	0 (0)	0 (0)	0 (0)
6	0 (0)	0 (0)	0 (0)
7	2 (25)	0 (0)	2 (18.2)
8	0 (0)	0 (0)	0 (0)
Total	8 (100)	3 (100)	11 (100.0)

**Table 3:** Health-related quality of life utility scores - summary statistics.

	**Study Group**		
	**<5 years old**	**≥5 years old**	**All patients**
N	8	3	11
mean	0.414 (severe)	0.309 (severe)	0.390 (severe)
Standard deviation	0.3990	0.0344	0.3367
Minimum	−0.188	0.295	−0.188
maximum	0.879	0.363	0.879

### Secondary analyses

Each of the under five-year-old children had a unique health state vector and 6 out of 8 were in the category of severe disability (Table 4), while all 3 subjects in the older group had unique health state vectors in the category of severe disability (Table 5). In the younger age group, 3 attributes had prevalences over 80 % for some morbidity: Ambulation (100 %), Emotion (88 %) and Pain and Discomfort (100 %). These same attributes have less than 50 % prevalence of moderate or severe morbidity (Table 6).

**Table 4:** Under five-year-old group - health state vectors, HRQL scores and disability category.

	HuPS Attribute Levels								HRQL Score	Disability Category
Subject ID	Vision	Hearing	Speech	Ambulation	Dexterity	Emotion	Cognition	Pain & Discomfort		
3	1	1	1	2	1	2	1	2	0.82	moderate
8	1	1	1	2	1	1	1	2	0.88	moderate
9	1	1	1	2	1	3	1	2	0.68	severe
10	1	1	2	4	1	2	1	2	0.53	severe
12	5	2	3	3	1	4	4	4	−0.11	severe
16	5	1	4	4	4	3	4	3	−0.19	severe
17	1	1	1	4	1	3	1	3	0.29	severe
20	1	1	1	2	1	3	2	3	0.41	severe
Subjects *n* = 8										

HRQL: Health-related quality of life; HuPS: Health Utilities Pre-School.

**Table 5:** Over five-year-old group - health state vectors, HRQL scores and disability category.

	HuPS Attribute Levels								HRQL Score	Disability Category
Subject ID	Vision	Hearing	Speech	Ambulation	Dexterity	Emotion	Cognition	Pain & Discomfort		
7	1	1	1	2	1	4	1	3	0.36	severe
15	1	1	4	4	1	2	1	3	0.32	severe
18	1	1	1	2	1	2	1	5	0.30	severe
Subjects *n* = 3										

HRQL: Health-related quality of life; HUI3: Health Utilities Index 3.

**Table 6:** Under five-year-old group - frequency of attribute levels.

	HuPS Attribute							
Attribute Level	Vision	Hearing	Speech	Ambulation	Dexterity	Emotion	Cognition	Pain & Discomfort
1	6 (75.0 %)	7 (87.5 %)	5 (62.5 %)	–	7 (87.5 %)	1 (12.5 %)	5 (62.5 %)	–
2	–	1 (12.5 %)	1 (12.5 %)	4 (50.0 %)	–	2 (25.0 %)	1 (12.5 %)	4 (50.0 %)
3	**–**	**–**	**1 (12.5 %)**	**1 (12.5 %)**	**–**	**4 (50.0 %)**	**–**	**3 (37.5 %)**
4	**–**	**–**	**1 (12.5 %)**	**3 (37.5 %)**	**1 (12.5 %)**	**1 (12.5 %)**	**2 (25.0 %)**	**1 (12.5 %)**
5	**2 (25.0 %)**	**–**	**n/a**	**n/a**	**n/a**	**n/a**	**n/a**	**n/a**
6	**–**	**n/a**	**n/a**	**n/a**	**n/a**	**n/a**	**n/a**	**n/a**
Total (n)	8 (100 %)	8 (100 %)	8 (100 %)	8 (100 %)	8 (100 %)	8 (100 %)	8 (100 %)	8 (100 %)
% subjects with some morbidity	25.0	12.5	37.5	100.0	12.5	87.5	37.5	100.0
% subjects with moderate or severe morbidity	25	0	25	50	12.5	62.5	25	50

HuPS: Health Utilities Pre-School; -: zero observations; n/a – not applicable (not defined); **bold**: moderate or severe level of disability.

## Discussion

The results of this study show a high burden of morbidity at diagnosis, often equating to severe disability, in children with advanced/high-risk neuroblastoma. In two under five-year-old children this burden was indicative of states of health worse than being dead.

In a prospective cross-sectional study of 19 children with advanced neuroblastomas in Canada, a predecessor of HuPS (CHSCS-PS) was administered at ten timepoints beginning before diagnosis.^20^ The highest mean disability scores were in a group of nine children who were assessed immediately after diagnosis, with the main burdens of morbidity related to mobility, self-care, emotion and pain. These findings are very similar to those of the study reported here. In a secondary analysis of the Canadian data, there was high inter-observer agreement between nurses and parents with an intra-class correlation of 0.86 for overall disability scores.^21^

The current pilot study, even though the sample size was small, recruited subjects from multiple institutions to measure their HRQL with an instrument designed for use in the most prevalent age group and to provide related utility scores. As expressed by Furlong et al.,^13^ “HuPS scores of HRQL are reliable, valid and interpretable. HuPS provides scores to inform important policy decisions considering the effectiveness and cost-effectiveness of healthcare services and the comprehensive health of populations. HuPS measurements are commensurate and continuous with those of HUI3 for group analyses.”

Nathan et al. noted that “Net effectiveness, using utility scores weighted by duration to calculate quality-adjusted life years (QALYs), forms the denominator in cost-utility economic evaluations. Cost-utility economic evaluations are used to inform decisions about alternative treatment protocols, given the health states, expected risk of relapse, and survival rates of the alternatives.”^20^ A cost-utility approach has been reported to compare health effects and inpatient/outpatient costs of acute lymphoblastic leukemia treatment in children over two years of follow up in seven centers in Canada, Italy and the United States. ^22^ Importantly, discounting and sensitivity testing were applied to both QALYs and costs, the latter from a hospital perspective. ^23^ Although there are few reports of this type from LMICs, a particularly valuable one has come from the national center in El Salvador that cares for children with cancer. ^24^ This report incorporated the principle of discounting and sensitivity analysis with a DALYs approach to analysis of cost-effectiveness, determining that the treatment of children in El Salvador was “very cost effective” using the World Health Organization Choosing Interventions that are Cost-Effective criterion of < 1 times the Gross Domestic Product per capita,^25^ even when late effects and early mortality are incorporated. The authors were careful to point out the difference between cost-effectiveness and affordability; the average cost per year for each newly diagnosed child (US$ 28,717) is ten times higher than a recent estimate of annual health expenditure per capita (US$ 280) in El Salvador.

## Conclusions

The results of the current study will help to set research priorities for subsequent investigations and provide a basis to improve supportive care of children with high-risk neuroblastoma. It will be especially informative to have a study of a large number of children receiving a common treatment protocol with measurement of HRQL at consistent points in their therapy. The availability of the HuPS instrument will allow such measurement in the great majority of these children and open the prospective of studies of the economics of their care.

## Funding

This work was supported by the International Initiative for Pediatrics and Nutrition (IIPAN).

## CRediT authorship contribution statement

**Karina Viani:** Conceptualization, Data curation, Funding acquisition, Investigation, Methodology, Project administration, Visualization, Writing – original draft, Writing – review & editing. **William Furlong:** Conceptualization, Formal analysis, Funding acquisition, Investigation, Methodology, Resources, Software, Validation, Visualization, Writing – original draft, Writing – review & editing. **Vicente Odone Filho:** Funding acquisition, Investigation, Methodology, Visualization, Writing – review & editing. **Mariana dos Santos Murra:** Data curation, Investigation, Visualization, Writing – review & editing. **Juliana Moura Nabarrete:** Data curation, Investigation, Visualization, Writing – review & editing. **Elena Ladas:** Funding acquisition, Methodology, Resources, Validation, Visualization, Writing – review & editing. **Ronald Duncan Barr:** Conceptualization, Formal analysis, Funding acquisition, Investigation, Methodology, Supervision, Validation, Visualization, Writing – original draft, Writing – review & editing.

## Conflicts of interest

The authors have no competing interests to declare.
